# The association between urinary phthalate metabolites and serum thyroid function in US adolescents

**DOI:** 10.1038/s41598-023-38644-2

**Published:** 2023-07-18

**Authors:** Shi-ting Xiang, Yuhan Cao, Jie Dong, Chao Li, Jun Qiu, Xun Li

**Affiliations:** 1 Hunan Children’s Research Institute (HCRI), Hunan Children’s Hospital, Ziyuan RD 86, Changsha, 410007 China; 2 Department of Occupational and Environmental Health, Xiangya School of Public Health, Central South University, Xiangya RD 110, Changsha, 410078 China; 3 Department of Epidemiology and Medical Statistics, Xiangya School of Public Health, Central South University, Xiangya RD 110, Changsha, 410078 China

**Keywords:** Biomarkers, Endocrinology

## Abstract

The aim was to investigate the association between mixed exposure to phthalates and serum thyroid function among US adolescents. The study used 2007–2008 survey data from the National Health and Nutrition Examination Survey (NHANES). Data on urinary phthalates metabolites and serum thyroid function indicators were collected. The weighted multivariable linear regression models and Bayesian kernel machine regression (BKMR) analyses were used to analyze the relationship between phthalates metabolites and thyroid function. A total of 356 adolescents aged 12–19 years were included in the analysis. Linear regression models showed that mono-(carboxyisoctyl) phthalate (MCOP) was positively correlated with total triiodothyronine (TT3) (β = 0.045, 95% confidence interval [CI] 0.022, 0.068) and thyroid stimulating hormone (TSH) (β = 0.1461, 95% CI 0.059, 0.232), while mono-(carboxyisononyl) phthalate (MCNP) was negatively correlated with TSH (β = − 0.119, 95% CI − 0.196, − 0.042). BKMR analyses showed phthalate metabolites mixtures have significantly positive overall effect on TT3. Exposure to phthalate mixtures might be positively correlated with increased TT3 serum level in US adolescents. The study provided evidence for the association between mixed phthalates exposure and thyroid health in adolescent population.

## Introduction

As the most important endocrine organ in the human body, thyroid plays an important role in promoting individual development and intellectual maturity^1^. Disruption of thyroid function, even with relatively minor changes, can lead to permanent growth defects and neurocognitive impairment^2^. The main indicators of thyroid function are thyroid stimulating hormone (TSH), total thyroxine (TT4), total triiodothyronine (TT3), free thyroxine (FT4), and free triiodothyronine (FT3). Thyroid hormones are regulated by hypothalamic-pituitary-thyroid (HPT) axis feedback to keep homeostasis^3^. Hyperthyroidism and hypothyroidism are common diseases affecting people’s health worldwide, with 0.2%–1.3% of people in iodine-sufficient areas suffering from overt hyperthyroidism and 0.2%–5.3% suffering from overt hypothyroidism^4^. In addition to iodine nutrition, ageing, smoking status, genetic susceptibility, and ethnicity, studies have found that endocrine disruptors can interfere with the HPT axis, alter thyroid hormone homeostasis, and ultimately affect the normal physiological function of thyroid^4,5^.

Phthalates are a class of diesters of 1, 2-phthalic acid, which have the ability to interfere with endocrine system. They are mainly used as plasticizer to increase the flexibility and durability of plastic products, such as toys, food packaging materials, and medical instruments. It can also be used as the carrier substrate for aroma components in personal care products, such as various cosmetics and washing products^6^. Phthalates are widely used in industrial production and daily life. As phthalates are usually bonded to polymers with non-chemical bonds, they are often released from plastic products into the surrounding environment and can be detected in the environment and human body, thus becoming an emerging chemical of concern^7^.

Increasing studies have shown that certain phthalate may alter thyroid function. For example, animal and human studies have shown that some phthalates may reduce T4 and T3 concentrations in pregnant women and children, antagonize the binding of T3 to thyroid receptor-β, reduce cell uptake of T3, and affect transcription of sodium-iodine transporters^8,9^. Meanwhile, a meta-analyse has shown a significant association between the exposure of diethylhexyl phthalate (DEHP) metabolites and the function of the HPT axis^10^. A recent study demonstrated that exposure to overall phthalates was associated with elevated levels of TT3 and FT4 in adults^11^. They also found that phthalate metabolites with different molecular weight showed opposite associations with thyroid hormones. Kyoung-Nam et al. studied the relationship between early life phthalates exposure and thyroid function tested at 6 years of age^12^. They found that phthalates exposure was associated with lower TSH and higher T3. Moreover, one study by Zheng et al. has revealed that exposure to phthalates may affect thyroid autoimmunity in underweight pregnant women during early pregnancy^13^. However, limited studies have explored the relationship between the exposures to phthalate metabolites mixtures with thyroid function measures in adolescents. This is particularly relevant, since the mixed toxicity effects induced and their interrelationships may differ from exposure to a single chemical^14,15^. In addition, puberty is a critical period of growth and development, early prevention and intervention can reduce profound adverse and irreversible effects in adulthood.

Therefore, to better understand the environmental determinants of adolescent thyroid hormones, the objective of present study was to investigate the association between mixed phthalates exposure and serum thyroid function in US adolescents. The study used data from a representative national sample of the National Health and Nutrition Examination Survey (NHANES). We used urinary phthalate metabolite concentrations as biomarker of internal exposure and TSH, FT4, TT4, FT3, and TT3 as indicators of thyroid function. Based on the Bayesian kernel machine regression (BKMR) model, we evaluated the association between mixed exposure to phthalate metabolites and serum thyroid function among adolescents.

## Materials and methods

### Study design and population

The analysis used data from the National Health and Nutrition Examination Survey (NHANES), whose design and sampling methods can be found in detail elsewhere^16^. In short, NHANES is a population-based cross-sectional survey designed to gather information about the health and nutrition of the U.S. household population. Each year, the project surveys a nationally representative sample of about 5000 people in counties across the country. The NHANES interview section covers demographic, socioeconomic, diet and health-related issues. Physical examination includes physiological measurement, laboratory examination and other contents. The study used data from a 2007–2008 survey. A total of 1210 participants aged 12 to19 completed the 2007–2008 survey, of which 356 participants received both phthalates and thyroid function tests and were included as the final sample.

### Assessments of phthalate metabolites, iodine, and thyroid function

Urine and blood specimens are collected at the mobile exam center when participants undergo a physical examination. They are then stored at − 20 °C until analysis. Urinary iodine concentration was determined by Inductively Coupled Plasma Dynamic Reaction Cell Mass Spectroscopy (ICP-DRC-MS). Urinary phthalate metabolites were quantitatively determined by high performance liquid chromatography-electrospray ionization-tandem mass spectrometry (HPLC-ESI-MS/MS)^17^. Eleven phthalate metabolites were included for statistical stability because they were detectable in more than 70% of the samples, including mono-(carboxyisononyl) phthalate (MCNP), mono-(carboxyisoctyl) phthalate (MCOP), mono-n-butyl phthalate (MnBP), mono-(3-carboxypropyl) phthalate (MCPP), mono-ethyl phthalate (MEP), mono-isobutyl phthalate (MiBP), mono-benzyl phthalate (MBzP), mono-2-ethyl-5-carboxypentyl phthalate (MECPP), mono-(2-ethyl-5-hydroxyhexyl) phthalate (MEHHP), mono-(2-ethylhexyl phthalate (MEHP), and mono-(2-ethyl-5-oxohexyl) phthalate (MEOHP). Sample concentration below the detection limits (LOD) was determined as the LOD/√2. Thyroid blood tests consisted a series of immunoenzyme assays, including total and free thyroxine (TT4/FT4), total and free triiodothyronine (TT3/FT3), and thyroid stimulating hormone (TSH).

### Covariates

According to previous studies^18,19^, we included eight covariates from the 2007–2008 survey, including age, gender, race, education, body mass index (BMI), energy, protein intake, and urinary iodine. The weight (kg) and height (m^2^) measured during physical examination were used to calculate the BMI. Energy and protein intake data were measured through face-to-face interviews in which they recalled their food intake over a 24-h period.

### Statistical analysis

Continuous data were described as mean and *SD* for normal distribution or median (interquartile range) for skewed distribution. Categorical data were described as numbers and percentages. We used urinary creatinine to adjust the concentrations of phthalate metabolites and urine iodine to adjust for variations in urine dilution. We used Spearman’s rank coefficients to evaluate the correlations of each pair of phthalate metabolites. The weighted multivariable linear regression models were applied to assess the association of phthalate metabolites with thyroid function. In addition, we conducted age and gender groups stratification analyses. Urinary phthalate metabolites, iodine, and serum thyroid function indicators were natural log transformed to improve the normality of the distributions. We used phthalate-specific subsample weight to account for the complex, multistage, probability sampling design of NHANES. We used false discovery rate (FDR) to correct *P* values and controlled the occurrence of class I errors. All models were adjusted for age, gender, race, education, BMI, energy, protein intake, and urinary iodine. Median values were used to fill in missing data on BMI (n = 4), energy (n = 10), protein intake (n = 10), and urinary iodine (n = 21).

BKMR analysis was conducted using the “bkmr package” for R version 3.5.1 to explore the health effects of mixed exposure to phthalates. It implements a Markov chain Monte Carlo (MCMC) algorithm with 1000 iterations^20^. First, BKMR studies the cumulative toxic effects of mixtures, by comparing the estimate of the exposure–response function when mixtures exposed to specific quantiles with those at the 50th percentile. Subsequently, the univariate relationship between each exposure and the outcome is obtained, where all of the other exposures are fixed to median values. Finally, in order to investigate whether the phthalate metabolite pairs interact with each other, the exposure–response function of a single exposure is investigated when the second exposure is fixed at different quantiles. BKMR models were adjusted for age, gender, race, education, BMI, energy, protein intake, and urinary iodine. All statistical analyses were performed using SPSS version 22.0 and R version 3.5.1. The significance level was set at *P* < 0.05 (two-sided).

### Ethics approval and consent to participate

The study was performed according to the Declaration of Helsinki. Informed written consent was provided from each participant or their proxy respondents. NHANES obtained ethics approval from the NCHS Research Ethics Review Board (Continuation of Protocol #2005-06).

## Results

### Descriptive statistics

The descriptive characteristics of 356 participants are presented in Table 1. A total of 52.0% of the subjects were male, and the average age of the included population was 15.59 years old. The mean BMI, energy and protein intake were 23.97 kg/m^2^, 2092.46 kcal and 76.22 g, respectively. In total, 32.9% of the participants were Non-hispanic whites. Overall, 83.4% of the participants had less than high school education level. The median urinary iodine (creatinine-corrected) was 124.41 ug/g. The median concentration of TT3, FT3, TT4, FT4, and TSH were 126.00 (112.00, 143.75) ng/dl, 3.60 (3.30, 3.90) pg/ml, 7.30 (6.60, 8.40) ug/dl, 0.80 (0.70, 0.90) ng/dl, and 2.06 (1.43, 3.27) uIU/ml.

**Table 1:** Characteristics of the participants in NHANES 2007–2008 (N = 356).

Characteristics	mean ± s or N(%) or median (P25, P75)
Age(years)	15.59 ± 2.26
BMI(kg/m^2^)	23.97 ± 6.24
Energy(kcal)	2092.46 ± 948.594
Protein intake(g)	76.22 ± 40.69
Gender	
Male	185 (52.0)
Female	171 (48.0)
Race	
Non-hispanic whites	117 (32.9)
Non-hispanic blacks	91 (25.6)
Mexican Americans	82 (23.0)
Other/Mixed race	66 (18.5)
Education level	
Less than high school	297 (83.4)
High School graduation or GED	28 (7.9)
College or higher	31 (8.7)
Urinary iodine (ug/g Cr)	124.41 (72.96, 219.70)
TT3 (ng/dl)	126.00 (112.00, 143.75)
FT3 (pg/ml)	3.60 (3.30, 3.90)
TT4 (ug/dl)	7.30 (6.60, 8.40)
FT4 (ng/dl)	0.80 (0.70, 0.90)
TSH (uIU/ml)	1.43 (0.99, 2.06)

Continuous data were described as mean ± SD (normal distribution) or median with 25–75 percentile (skewed distribution); categorical data were described as n (%). A total of 4, 10, 10, and 21 participants had missing information on BMI, energy, protein intake, and urinary iodine, respectively.

The limit of detection (LOD), detection rate and distribution of urinary phthalate metabolites concentration are shown in Table 2. The lowest and highest detection rates were 71.3% and 100%, respectively. MEP had the highest median concentrations and the lowest for MCNP.

**Table 2:** Levels of urinary phthalate metabolites (μg/mmol Cr, N = 356).

Phthalate metabolites	LOD	≥ LOD%	GM	P_5_	P_25_	P_50_	P_75_	P_95_
MCNP	0.5	92.4	0.25	0.07	0.15	0.22	0.38	1.04
MCOP	0.7	98.3	0.73	0.16	0.34	0.61	1.27	6.03
MnBP	0.6	99.7	2.30	0.60	1.45	2.39	3.81	8.03
MCPP	0.2	98.3	0.32	0.08	0.18	0.30	0.54	1.67
MEP	0.462	100.0	10.53	2.02	4.54	9.34	21.79	84.20
MiBP	0.3	99.4	0.93	0.22	0.57	0.99	1.57	3.03
MBzP	0.216	99.2	0.96	0.14	0.56	1.00	1.82	5.16
MEHP	1.1	71.3	0.28	0.05	0.11	0.25	0.51	3.37
MEOHP	1.848	100.0	1.45	0.28	0.67	1.29	2.68	14.94
MEHHP	0.7	100.0	2.56	0.52	1.18	2.30	4.75	27.18
MECPP	0.5	100.0	3.84	0.93	1.89	3.22	6.89	29.30

### Correlations between phthalates

The Spearman’s rank coefficients between creatinine-corrected urinary phthalate metabolites are shown in Table 3. Most pairs have positive correlations. In general, the closer the correlation coefficient is to 1, the better the correlation is. When the correlation coefficient is greater than 0.3, the correlation is worth considering. Based on this, most pairs had positive correlations. For example, in this study, MCPP was positively correlated with MCOP, and MnBP was positively correlated with MiBP. Some pairs, although *P* < 0.01, were not considered to be correlated due to the small correlation coefficient.

**Table 3:** Correlation coefficient between different phthalate metabolites (r-value) (N = 356).

	∑DEHP	MCNP	MCOP	MnBP	MCPP	MEP	MiBP	MBzP
∑DEHP	1	0.293**	0.553**	0.388**	0.523**	0.071	0.365**	0.294**
MCNP	–	1	0.538**	0.172**	0.380**	0.033	0.167**	0.158**
MCOP	–	–	1	0.275**	0.650**	0.016	0.279**	0.307**
MnBP	–	–	–	1	0.398**	0.112*	0.662**	0.588**
MCPP	–	–	–	–	1	0.020	0.298**	0.397**
MEP	–	–	–	–	–	1	0.112*	0.011
MiBP	–	–	–	–	–	–	1	0.467**
MBzP	–	–	–	–	–	–	–	1

*P < 0.05, **P < 0.01.

The total concentration of di (2-ethylhexyl) phthalate (∑DEHP) was estimated using the sum of the molar concentrations of MECPP, MEHP, MEHHP, and MEOHP and multiplied by the molar weight of MEHP (MW = 278).

### Associations between phthalate metabolites and thyriod parameters

We used the weighted multivariable linear regression models to examine the associations between phthalate metabolites and serum thyroid function (Table 4). The results showed that ∑DEHP (β = 0.026, 95% confidence interval [CI]: 0.003, 0.048) and MCOP (β = 0.045, 95% CI 0.022, 0.068) were positively correlated with TT3, and MCNP (β = − 0.023, 95% CI − 0.043, − 0.004) and MCPP (β = − 0.029, 95% CI − 0.055, − 0.002) were negativity correlated with TT3. There was also significant positive association between ∑DEHP (β = 0.032, 95% CI 0.003, 0.06) and TT4, while inverse association between MCPP (β = − 0.048, 95% CI − 0.085, − 0.011) and TT4. A significant positive association was also observed between MCOP (β = 0.1461, 95% CI 0.059, 0.232) and TSH, while a significant negative correlation was found between MCNP (β = − 0.119, 95% CI − 0.196, − 0.042) and TSH. After further correcting *P* values, we only found that exposure to MCOP was positively correlated with TT3 and TSH, and MCNP was negatively correlated with TSH (all *P*_FDR_ < 0.05). When stratified by age, we found that MBzP was negatively correlated with TT3 and TSH, and MnBP was positively correlated with TT3 in the 18–19 years old group (Table S1). In analysis stratified by sex, MCPP was inversely associated with TT4 in males (Table S2). The associations between urinary MCOP and MCNP and TT3 and TSH were consistent between age groups and sex groups.

**Table 4:** Multivariable adjusted relationship between phthalate metabolites and thyroid indicators.

	∑DEHP	MCNP	MCOP	MnBP	MCPP	MEP	MiBP	MBzP
TT3 (ng/dl)								
β (95% CI)	0.026 (0.003, 0.048) *	− 0.023 (− 0.043, − 0.004) *	0.045 (0.022, 0.068) **	− 0.003 (− 0.045, 0.040)	− 0.029 (− 0.055, − 0.002) *	− 0.010 (− 0.026, 0.007)	0.008 (− 0.028, 0.044)	− 0.009 (− 0.031, 0.014)
*P*_FDR_	0.163	0.135	0.008**	0.949	0.163	0.669	0.949	0.930
FT3 (pg/ml)								
β (95% CI)	0.004 (− 0.009, 0.018)	− 0.001 (− 0.015, 0.013)	0.000 (− 0.014, 0.014)	0.001 (− 0.024, 0.027)	0.006 (− 0.010, 0.023)	− 0.013 (− 0.025, 0.000)*	0.003 (− 0.018, 0.025)	0.003 (− 0.012, 0.019)
*P*_FDR_	0.949	0.949	0.995	0.949	0.930	0.207	0.949	0.949
TT4 (ug/dl)								
β (95% CI)	0.032 (0.003, 0.060)*	0.006 (− 0.021, 0.033)	0.002 (− 0.027, 0.030)	0.003 (− 0.044, 0.050)	− 0.048 (− 0.085,− 0.011)*	0.005 (− 0.012, 0.022)	0.004 (− 0.030, 0.037)	− 0.007 (− 0.034, 0.019)
*P*_FDR_	0.163	0.949	0.949	0.949	0.105	0.949	0.949	0.949
FT4 (ng/dl)								
β (95% CI)	0.015 (− 0.007, 0.037)	0.001 (− 0.024, 0.027)	− 0.010 (− 0.037, 0.016)	− 0.031 (− 0.076, 0.014)	− 0.019 (− 0.052, 0.013)	0.001 (− 0.016, 0.018)	0.013 (− 0.021, 0.048)	0.006 (− 0.020, 0.032)
*P*_FDR_	0.619	0.949	0.930	0.619	0.669	0.949	0.930	0.949
TSH(uIU/ml)								
β (95% CI)	0.043 (− 0.026, 0.112)	− 0.119 (− 0.196, − 0.042)*	0.146 (0.059, 0.232)*	− 0.082 (− 0.229, 0.066)	− 0.084 (− 0.183, 0.015)	− 0.005 (− 0.072, 0.062	0.022 (− 0.093, 0.138)	0.004 (− 0.084, 0.092)
*P*_FDR_	0.669	0.034*	0.021*	0.695	0.396	0.949	0.949	0.949

*P < 0.05, **P < 0.01.

The total concentration of di (2-ethylhexyl) phthalate (∑DEHP) was estimated using the sum of the molar concentrations of MECPP, MEHP, MEHHP, and MEOHP and multiplied by the molar weight of MEHP (MW = 278).* P*_FDR_ is the *P* value adjusted by the method of Benjamini–Hochberg false discovery rate (FDR) correction to adjust for multiple testing. All models were adjusted for age, gender, race, education, BMI, energy, protein intake, and urinary iodine.

### BKMR analyses

**Figure 1:**
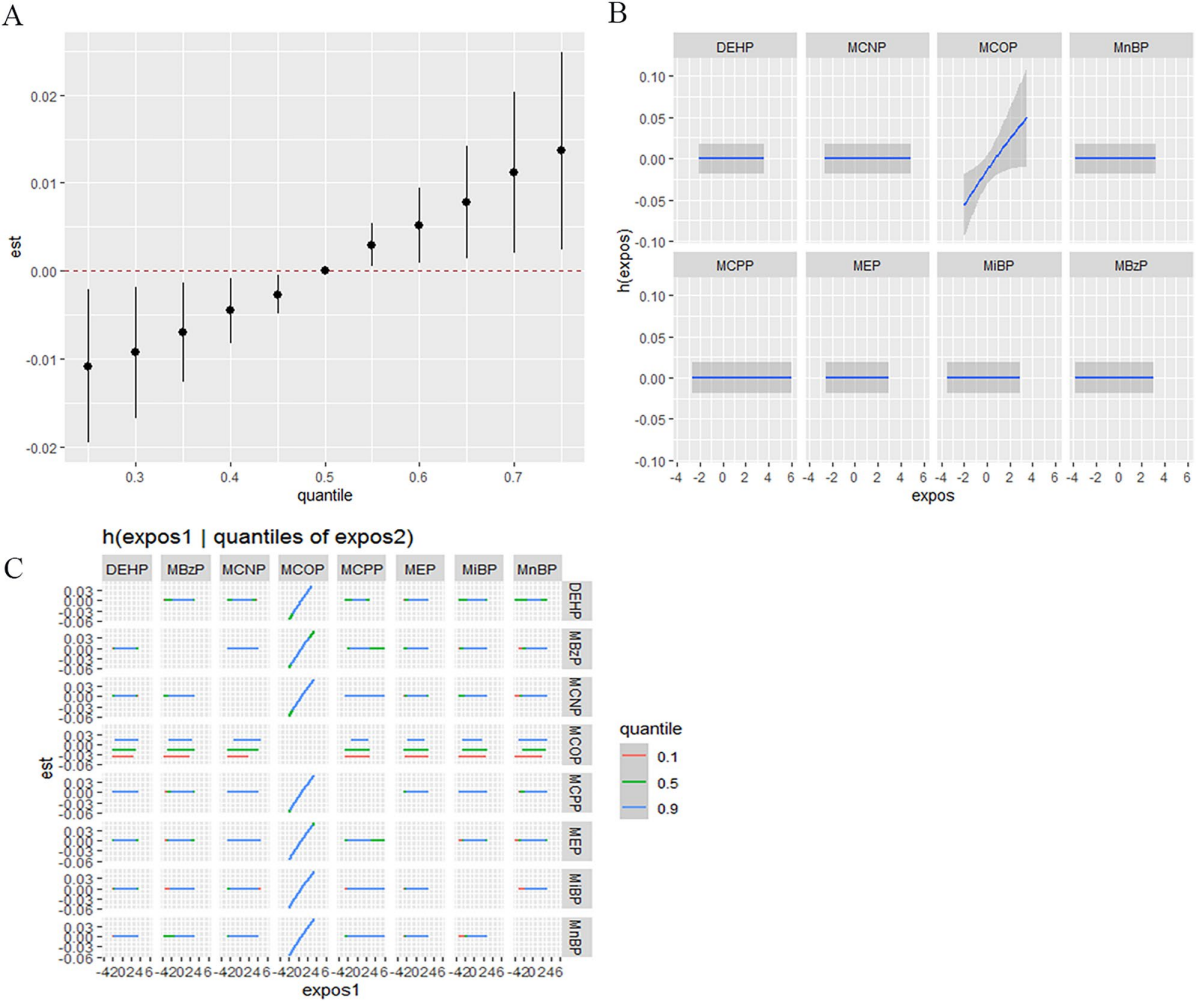
(**A**) Cumulative effect of the phthalate metabolites mixture and TT3. (**B**) Univariate relationship between each phthalate metabolite and TT3, with other metabolites fixed at median values. (**C**) Bivariate exposure–response function. The model is adjusted for age, gender, race, education, BMI, energy, protein intake, and urinary iodine.

**Figure 2:**
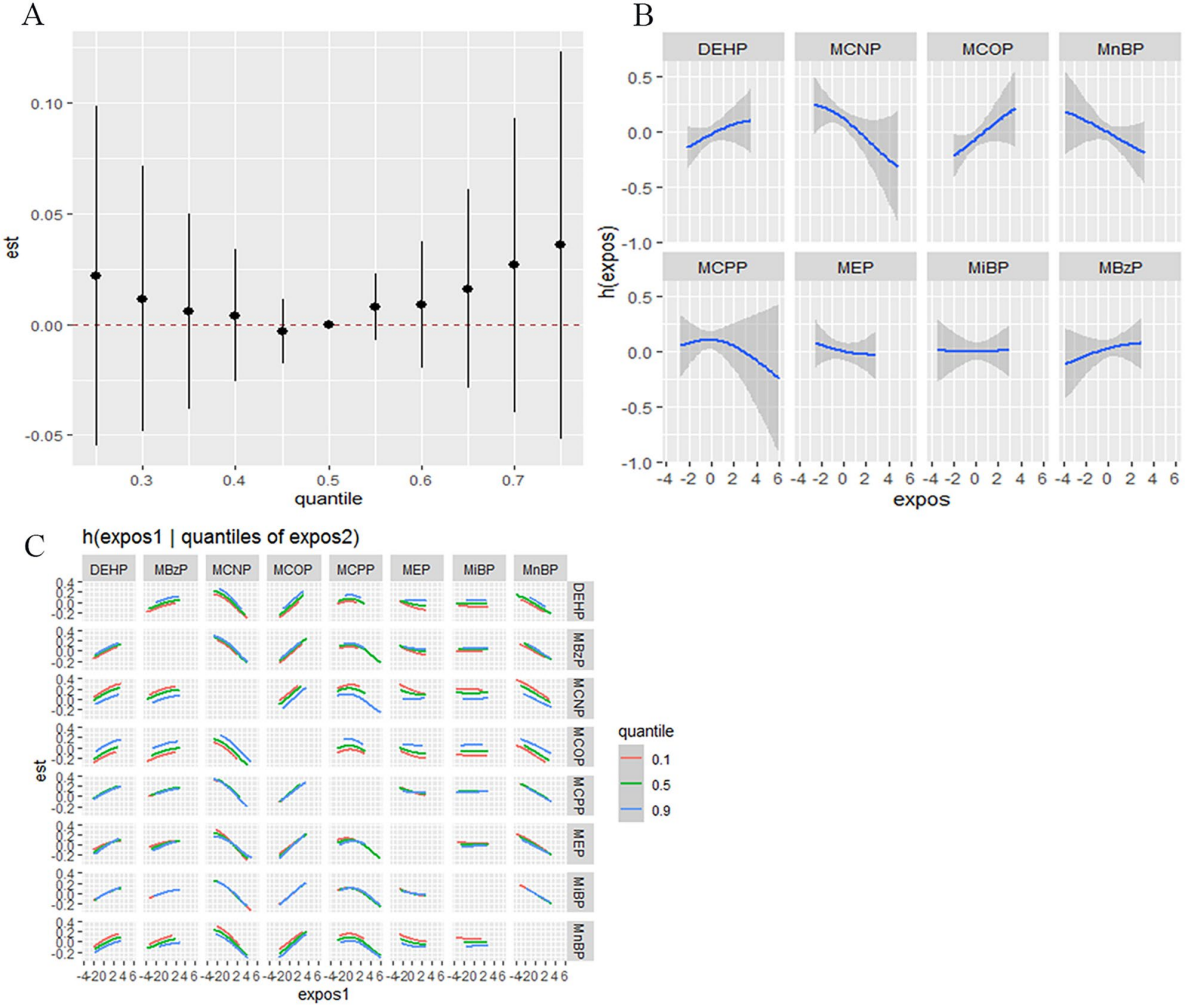
(**A**) Cumulative effect of the phthalate metabolites mixture and TSH. (**B**) Univariate relationship between each phthalate metabolite and TSH, with other metabolites fixed at median values. (**C**) Bivariate exposure–response function. The model is adjusted for age, gender, race, education, BMI, energy, protein intake, and urinary iodine.

Given the limitations associated with linearity and interaction in regression analysis, we use BKMR methods to further explore the effects of phthalates mixtures. Figure 1A showed that as cumulative levels across all phthalate metabolites increased, the TT3 concentration increased. We can see that phthalate metabolites have cumulative toxicity. Figure 1B showed that the concentration of MCOP and TT3 presented a linear relationship. Figure 1C showed that the slopes of MCOP exposure–response function was similar, indicating no interaction between MCOP and other phthalates. Figure 2A showed that there were no significant changes in TSH concentration when estimating the cumulative effect of combined exposure to phthalate metabolites at different thresholds. Figure 2B showed a linear relationship between MCOP, MCNP and TSH. Figure 2C showed that the slope of MCOP exposure–response function was similar to that of MCNP in different quantiles, indicating no interaction between the two.

## Discussion

This is the first study to evaluate the overall effects of phthalates mixtures exposure on measures of thyroid function among US adolescents. In this cross-sectional study, we found evidence that exposure to MCOP was positively correlated with TT3 and TSH, and MCNP was negatively correlated with TSH among adolescents. The result of BKMR analysis was similar to that of linear regression model. A significant positive relationship between the phthalate mixtures exposure and TT3 was revealed.

T4 and T3 are thyroid hormones that are necessary for growth, neuronal development, reproduction, and regulation of energy metabolism. FT4 and FT3 are "free" forms of T4 and T3 that can enter cells to perform physiological functions, but in very small quantities. TT4 is the sum of T4 and FT4, and TT3 is the sum of T3 and FT3^21^. Elevated levels of TT4 and TT3 are indicators of hyperthyroidism, while decreased levels are primarily seen in hypothyroidism. Both excessively high or low levels of thyroid hormones can lead to potential adverse reactions in adolescents and adults. For example, the adverse effects associated with hyperthyroidism include weight loss, increased heart rate, nervousness and anxiety, and decreased bone density. Hypothyroidism is associated with adverse effects such as weight gain, fatigue, depression and mood swings, lack of concentration, and poor memory^22–25^.

A previous study^18^ studied 329 adolescents aged 12–19 years from the 2007–2008 NHANES reported that exposure to DEHP metabolites was positively associated with TT3 and MCPP was inversely associated with FT4 and TT4 among the adolescents aged 12–19 years. Although our study found similar linear correlations between ∑DEHP with TT3 and MCPP with TT4, these relationships were weakened when our models corrected for *P* values. Two possible reasons for this inconsistence may be that we used urinary creatinine to adjust phthalate metabolites concentrations and we didn’t exclude the subjects with a possible thyroid disease or with outlying values. The two statistical methods in this study indicated that MCOP was positively correlated with TT3. In addition, the mixed effect of phthalate mixtures on TT3 was statistically significant in the BKMR model, with MCOP being the main positive correlation factor. A series of studies have examined the relationship between phthalates and thyroid function in children, adults, and pregnant women. A longitudinal cohort study^26^ of 166 children aged 2–18 years showed MEHHP was positively associated with FT4 and MMP were negatively associated with T3, T4 and FT4. A previous study by Meeker et al.^18^ reported that adult urinary DEHP metabolites were significantly negatively correlated with TT4, FT4, and TT3, and positively correlated with TSH. A population-based prospective cohort study^27^ of 1996 pregnant women found that higher DEHP was associated with lower FT4, while higher DINP metabolites were associated with lower TT4. They also reported that a higher diisononyl cyclohexane dicarboxylate (DINCH) metabolite concentration was associated with higher TT3. While studies have suggested that certain phthalates may damage the thyroid, their conclusions, such as which phthalates alter which specific thyroid hormones, were inconsistent. The differences in results between different populations may come from the timing and dose of exposure to phthalates.

TSH is an important hormone secreted by the adenohypophysial gland that can stimulate the development, synthesis and secretion of thyroid cells^22^. Some studies ^[228,12]^have revealed that exposure to phthalates were negatively associated with TSH. For example, Kim et al.^12^ reported that MnBP and MEOHP exposure during pregnancy or at 4 years of age was associated with lower TSH. Our study found that exposure to MCOP was positively correlated with TSH, and MCNP was negatively correlated with TSH. MCOP and MCNP are metabolites of DINP and DIDP, respectively. DINP and DIDP are both long-branched high molecular weight phthalates^29^. A previous experimental study^30^ reported that DINP and DIDP have no cytotoxicity at certain concentrations and can significantly regulate iodine uptake activity, which is of great significance for thyroid hormone synthesis. Another animal study^31^ reported that DINP can aggravate thyroid autoimmunity in rats. Human evidence on the association between DINP and DIDP and TSH is rather limited. Future studies are necessary to confirm these associations and elucidate the biological mechanisms underlying these differences.

In this study, we investigated the associations between mixed phthalate metabolites exposure and thyroid function in adolescents. The study will provide preliminary evidence for a relationship between mixed phthalates exposure and thyroid health in this population. We employed a novel statistical method BKMR, which is able to quantify and visualize the joint effects and interactions between different phthalate metabolites during exposure. This study has several limitations. First, because our study was based on data from a cross-sectional design, we were unable to establish a causal relationship between phthalate metabolite exposure and thyroid function. Secondly, because we didn’t have information on the subject's thyroid disease, we didn’t exclude the subjects with a possible thyroid disease. Third, we didn’t exclude the subjects with outlying values since thyroid hormone measurement is not sufficient to diagnose thyroid disease. And in our models, thyroid hormones indicators were natural log-transformed to reduce outlier effects. Fourth, phthalate metabolites concentration was measured with single-spot urine sample, which is less robust than multipoint urine.

Our study, based on NHANES survey data from 2007–2008, found that exposure to phthalate mixtures may be positively correlated with increased TT3 serum levels in US adolescents. Given the widespread presence of phthalate exposure and the critical period of adolescent development, this study has significant public health implications for understanding the association between phthalate mixtures and thyroid function.

## Supplementary Information

Supplementary Tables.

## Data Availability

The datasets generated and analysed during the current study are available in survey data and documentation of National Health and Nutrition Examination Survey (https://www.cdc.gov/nchs/nhanes/index.htm).

## References

[1] Tarım Ö (2011). Thyroid hormones and growth in health and disease. J. Clin. Res. Pediatr. Endocrinol..

[2] De Leo S, Lee SY, Braverman LE (2016). Hyperthyroidism. Lancet.

[3] Yorita Christensen KL (2013). Metals in blood and urine, and thyroid function among adults in the United States 2007–2008. Int. J. Hyg. Environ. Health.

[4] Taylor PN, Albrecht D, Scholz A, Gutierrez-Buey G, Lazarus JH, Dayan CM (2018). Global epidemiology of hyperthyroidism and hypothyroidism. Nat. Rev. Endocrinol..

[5] Yilmaz B, Terekeci H, Sandal S, Kelestimur F (2020). Endocrine disrupting chemicals: Exposure, effects on human health, mechanism of action, models for testing and strategies for prevention. Rev. Endocr. Metab. Disord..

[6] Wittassek M, Wiesmüller GA, Koch HM, Eckard R, Dobler L, Müller J (2007). Internal phthalate exposure over the last two decades–a retrospective human biomonitoring study. Int. J. Hyg. Environ. Health.

[7] Zhang YJ, Guo JL, Xue JC, Bai CL, Guo Y (2021). Phthalate metabolites: Characterization, toxicities, global distribution, and exposure assessment. Environ. Pollut..

[8] Braun JM (2017). Early-life exposure to EDCs: Role in childhood obesity and neurodevelopment. Nat. Rev. Endocrinol..

[9] Ghisari M, Bonefeld-Jorgensen EC (2009). Effects of plasticizers and their mixtures on estrogen receptor and thyroid hormone functions. Toxicol. Lett..

[10] Kim MJ, Moon S, Oh BC (2019). Association between diethylhexyl phthalate exposure and thyroid function: A meta-analysis. Thyroid.

[11] Donat-Vargas C, Perez-Carrascosa F, Gomez-Peña C, Mustieles V, Salcedo-Bellido I, Frederiksen H (2021). Associations of serum phthalate metabolites with thyroid hormones in GraMo cohort, Southern Spain. Environ. Pollut..

[12] Kim KN, Kim HY, Lim YH, Shin CH, Kim JI, Kim BN (2020). Prenatal and early childhood phthalate exposures and thyroid function among school-age children. Environ. Int..

[13] Yang Z, Zhang T, Shan D, Li L, Wang S, Li Y (2022). Associations between phthalate exposure and thyroid function in pregnant women during the first trimester. Ecotoxicol. Environ. Saf..

[14] Czarnota J, Gennings C, Colt JS, De Roos AJ, Cerhan JR, Severson RK (2015). Analysis of environmental chemical mixtures and non-hodgkin lymphoma risk in the NCI-SEER NHL study. Environ. Health Perspect..

[15] Zhang X, Guo N, Jin H, Liu R, Zhang Z, Cheng C (2022). Bisphenol A drives di(2-ethylhexyl) phthalate promoting thyroid tumorigenesis via regulating HDAC6/PTEN and c-MYC signaling. J. Hazard Mater..

[16] National Health and Nutrition Examination Survey. NHANES 2007–2008. http://wwwn.cdc.gov/nchs/nhanes/search/nhanes07_08.aspx

[17] NHANES. National Health and Nutrition Examination Survey. 2007–2008 LabMethods.http://www.cdc.gov/nchs/nhanes/nhanes2007-2008/lab_methods_07_08.htm

[18] Meeker JD, Ferguson KK (2011). Relationship between urinary phthalate and bisphenol A concentrations and serum thyroid measures in U.S. adults and adolescents from the National Health and Nutrition Examination Survey (NHANES) 2007–2008. Environ. Health Perspect..

[19] Sun Y, Xia PF, Korevaar TIM, Mustieles V, Zhang Y, Pan XF (2021). Relationship between blood trihalomethane concentrations and serum thyroid function measures in U.S.. Adults. Environ. Sci. Technol..

[20] Shah-Kulkarni S, Lee S, Jeong KS, Hong YC, Park H, Ha M (2020). Prenatal exposure to mixtures of heavy metals and neurodevelopment in infants at 6 months. Environ. Res..

[21] Garmendia Madariaga A, Santos Palacios S, Guillén-Grima F, Galofré JC (2014). The incidence and prevalence of thyroid dysfunction in Europe: A meta-analysis. J. Clin. Endocrinol. Metab..

[22] Cooper DS, Biondi B (2012). Subclinical thyroid disease. Lancet.

[23] Jabbar A, Pingitore A, Pearce SH, Zaman A, Iervasi G, Razvi S (2017). Thyroid hormones and cardiovascular disease. Nat. Rev. Cardiol..

[24] Knudsen N, Laurberg P, Rasmussen LB, Bülow I, Perrild H, Ovesen L, Jørgensen T (2005). Small differences in thyroid function may be important for body mass index and the occurrence of obesity in the population. J. Clin. Endocrinol. Metab..

[25] Biondi B, Palmieri EA, Fazio S, Cosco C, Nocera M, Saccà L (2000). Endogenous subclinical hyperthyroidism affects quality of life and cardiac morphology and function in young and middle-aged patients. J. Clin. Endocrinol. Metab..

[26] Huang PC, Chang WH, Wu MT, Chen ML, Wang IJ, Shih SF (2020). Characterization of phthalate exposure in relation to serum thyroid and growth hormones, and estimated daily intake levels in children exposed to phthalate-tainted products: A longitudinal cohort study. Environ. Pollut..

[27] Derakhshan A, Shu H, Broeren MAC, Lindh CH, Peeters RP, Kortenkamp A (2021). Association of phthalate exposure with thyroid function during pregnancy. Environ. Int..

[28] Romano ME, Eliot MN, Zoeller RT, Hoofnagle AN, Calafat AM, Karagas MR (2018). Maternal urinary phthalate metabolites during pregnancy and thyroid hormone concentrations in maternal and cord sera: The HOME Study. Int. J. Hyg. Environ. Health..

[29] Odebeatu CC, Taylor T, Fleming LE, Osborne JN (2019). Phthalates and asthma in children and adults: US NHANES 2007–2012. Environ. Sci. Pollut. Res. Int..

[30] Wenzel A, Franz C, Breous E, Loos U (2005). Modulation of iodide uptake by dialkyl phthalate plasticisers in FRTL-5 rat thyroid follicular cells. Mol. Cell Endocrinol..

[31] Duan J, Kang J, Deng T, Yang X, Chen M (2018). Exposure to DBP and high iodine aggravates autoimmune thyroid disease through increasing the levels of IL-17 and thyroid-binding globulin in wistar rats. Toxicol. Sci..

