# Supplementary material for: The association between urinary phthalate metabolites and serum thyroid function in US adolescents
Source: Sci Rep. 2023 Jul 18;13:11601. doi: 10.1038/s41598-023-38644-2 (PMC10354030; doi:10.1038/s41598-023-38644-2)
Supplement: Supplementary file 1 — Supplementary Tables. [file 41598_2023_38644_MOESM1_ESM.docx]

Supplemental file

Table S1. Association between phthalate metabolites and thyroid indicators stratified by Age groups

| Thyroid indicators / Phthalate metabolites | β (95% CI) | | |
| --- | --- | --- | --- |
|  | 12-14 years old(N=129) | 15-17 years old(N=137) | 18-19 years old(N=90) |
| TT3 (ng/dl) |  |  |  |
| ∑DEHP | 0.017(-0.019,0.052) | 0.046(0.009,0.083) | -0.002(-0.041,0.037) |
| MCNP | -0.033(-0.073,0.007) | -0.019(-0.047,0.009) | -0.065(-0.120,-0.010) |
| MCOP | 0.008(-0.035,0.051) | 0.061(0.016,0.106) | 0.051(0.007,0.095) |
| MnBP | 0.005(-0.078,0.088) | -0.039(-0.104,0.027) | **0.092(0.043,0.142)** |
| MCPP | -0.021(-0.081,0.040) | -0.051(-0.092,-0.010) | 0.005(-0.034,0.043) |
| MEP | -0.019(-0.055,0.017) | -0.021(-0.048,0.007) | -0.009(-0.032,0.013) |
| MiBP | 0.035(-0.027,0.097) | 0.030(-0.011,0.072) | -0.082(-0.139,-0.025) |
| MBzP | 0.015(-0.022,0.051) | -0.008(-0.042,0.025) | **-0.069(-0.110,-0.027)** |
| FT3(pg/ml) |  |  |  |
| ∑DEHP | 0.002(-0.016,0.019) | 0.006(-0.018,0.029) | -0.009(-0.043,0.025) |
| MCNP | -0.010(-0.031,0.012) | 0.016(-0.001,0.033) | -0.028(-0.081,0.025) |
| MCOP | 0.004(-0.025,0.032) | -0.021(-0.044,0.001) | 0.018(-0.018,0.055) |
| MnBP | 0.005(-0.034,0.045) | -0.003(-0.042,0.035) | 0.020(-0.039,0.079) |
| MCPP | -0.003(-0.038,0.033) | 0.000(-0.025,0.026) | 0.026(-0.005,0.058) |
| MEP | -0.010(-0.030,0.011) | -0.015(-0.031,0.001) | -0.015(-0.040,0.010) |
| MiBP | 0.021(-0.006,0.048) | -0.002(-0.034,0.030) | -0.001(-0.055,0.052) |
| MBzP | 0.008(-0.012,0.029) | 0.030(0.003,0.057) | -0.038(-0.077,0.002) |
| TT4 (ug/dl) |  |  |  |
| ∑DEHP | 0.029(-0.011,0.070) | 0.033(-0.010,0.076) | 0.006(-0.045,0.056) |
| MCNP | 0.044(-0.002,0.090) | -0.011(-0.040,0.018) | -0.050(-0.109,0.010) |
| MCOP | -0.045(-0.095,0.004) | 0.021(-0.025,0.067) | 0.037(-0.013,0.086) |
| MnBP | -0.027(-0.103,0.049) | -0.029(-0.093,0.035) | 0.108(0.029,0.187) |
| MCPP | -0.032(-0.094,0.030) | -0.063(-0.124,-0.002) | -0.028(-0.086,0.030) |
| MEP | 0.012(-0.026,0.050) | 0.002(-0.022,0.026) | -0.017(-0.048,0.013) |
| MiBP | -0.004(-0.053,0.045) | 0.010(-0.040,0.060) | 0.000(-0.083,0.083) |
| MBzP | 0.041(0.003,0.079) | -0.016(-0.055,0.022) | **-0.105(-0.168,-0.041)** |
| FT4(ng/dl) |  |  |  |
| ∑DEHP | -0.006(-0.046,0.033) | 0.016(-0.012,0.045) | 0.019(-0.024,0.061) |
| MCNP | 0.010(-0.034,0.054) | 0.018(-0.013,0.049) | -0.060(-0.113,-0.006) |
| MCOP | 0.037(-0.004,0.079) | -0.038(-0.075,-0.001) | 0.014(-0.033,0.062) |
| MnBP | -0.060(-0.130,0.009) | -0.077(-0.141,-0.012) | 0.020(-0.048,0.088) |
| MCPP | -0.062(-0.113,-0.011) | 0.027(-0.027,0.081) | -0.038(-0.083,0.006) |
| MEP | 0.008(-0.027,0.044) | 0.002(-0.018,0.022) | -0.014(-0.041,0.013) |
| MiBP | 0.034(-0.028,0.095) | 0.012(-0.033,0.058) | 0.022(-0.057,0.101) |
| MBzP | 0.038(0.005,0.070) | -0.012(-0.049,0.025) | -0.020(-0.071,0.030) |
| TSH(uIU/ml) |  |  |  |
| ∑DEHP | 0.090(-0.026,0.206) | 0.077(-0.036,0.190) | -0.014(-0.129,0.101) |
| MCNP | -0.110(-0.233,0.012) | -0.133(-0.253,-0.013) | -0.005(-0.179,0.169) |
| MCOP | 0.123(-0.007,0.253) | 0.160(0.013,0.307) | 0.103(-0.041,0.247) |
| MnBP | -0.177(-0.319,-0.034) | -0.099(-0.371,0.172) | 0.071(-0.181,0.323) |
| MCPP | -0.006(-0.146,0.134) | -0.133(-0.298,0.031) | -0.024(-0.190,0.143) |
| MEP | 0.064(-0.047,0.175) | -0.041(-0.127,0.044) | 0.018(-0.076,0.113) |
| MiBP | -0.017(-0.151,0.118) | 0.108(-0.074,0.291) | -0.176(-0.416,0.064) |
| MBzP | 0.023(-0.091,0.136) | -0.042(-0.206,0.122) | 0.146(-0.008,0.300) |

Values in bold are statistically significant *(P* _FDR_ < 0.05*)*

Note: The total concentration of di (2-ethylhexyl) phthalate (∑DEHP) was estimated using the sum of the molar concentrations of MECPP, MEHP, MEHHP, and MEOHP and multiplied by the molar weight of MEHP (MW=278). *P* _FDR_ is the *P* value adjusted by the method of Benjamini-Hochberg false discovery rate (FDR) correction to adjust for multiple testing. All models were adjusted for age, gender, race, education, BMI, energy, protein intake, and urinary iodine.

Table S2. Association between phthalate metabolites and thyroid indicators stratified by sex groups

| Thyroid indicators / Phthalate metabolites | β (95% CI) | |
| --- | --- | --- |
|  | Male(N=185) | Female(N=171) |
| TT3 (ng/dl) |  |  |
| ∑DEHP^a^ | 0.013(-0.019,0.045) | 0.033(0.001,0.065) |
| MCNP | -0.027(-0.047,-0.006) | -0.017(-0.054,0.019) |
| MCOP | **0.057(0.028,0.086)** | 0.033(-0.006,0.072) |
| MnBP | 0.021(-0.040,0.081) | -0.026(-0.094,0.043) |
| MCPP | -0.038(-0.071,-0.005) | -0.016(-0.064,0.031) |
| MEP | -0.005(-0.029,0.019) | -0.017(-0.043,0.009) |
| MiBP | -0.001(-0.057,0.056) | 0.012(-0.034,0.057) |
| MBzP | -0.004(-0.027,0.019) | -0.017(-0.062,0.027) |
| FT3(pg/ml) |  |  |
| ∑DEHP^a^ | 0.002(-0.018,0.023) | 0.001(-0.017,0.019) |
| MCNP | -0.003(-0.019,0.014) | -0.004(-0.027,0.020) |
| MCOP | 0.010(-0.011,0.030) | -0.013(-0.034,0.007) |
| MnBP | 0.003(-0.030,0.036) | 0.008(-0.027,0.044) |
| MCPP | 0.012(-0.009,0.032) | 0.006(-0.019,0.031) |
| MEP | 0.001(-0.015,0.017) | -0.029(-0.049,-0.009) |
| MiBP | -0.005(-0.033,0.023) | 0.003(-0.024,0.030) |
| MBzP | 0.001(-0.020,0.022) | 0.000(-0.024,0.025) |
| TT4 (ug/dl) |  |  |
| ∑DEHP^a^ | -0.002(-0.030,0.027) | 0.056(0.013,0.100) |
| MCNP | 0.016(-0.017,0.048) | -0.001(-0.043,0.040) |
| MCOP | 0.026(-0.009,0.060) | -0.014(-0.055,0.027) |
| MnBP | 0.040(-0.009,0.090) | -0.034(-0.110,0.043) |
| MCPP | **-0.072(-0.117,-0.026)** | -0.020(-0.072,0.032) |
| MEP | 0.014(-0.010,0.038) | -0.001(-0.028,0.026) |
| MiBP | -0.031(-0.069,0.006) | 0.029(-0.022,0.081) |
| MBzP | -0.001(-0.031,0.029) | -0.013(-0.056,0.030) |
| FT4(ng/dl) |  |  |
| ∑DEHP^a^ | 0.020(-0.014,0.053) | 0.014(-0.017,0.045) |
| MCNP | -0.004(-0.036,0.029) | 0.011(-0.028,0.050) |
| MCOP | 0.008(-0.028,0.045) | -0.026(-0.058,0.006) |
| MnBP | -0.012(-0.067,0.042) | -0.034(-0.105,0.036) |
| MCPP | -0.027(-0.071,0.018) | -0.011(-0.051,0.029) |
| MEP | 0.014(-0.008,0.036) | -0.010(-0.034,0.013) |
| MiBP | -0.007(-0.047,0.033) | 0.021(-0.031,0.073) |
| MBzP | 0.001(-0.030,0.032) | 0.005(-0.035,0.045) |
| TSH(uIU/ml) |  |  |
| ∑DEHP | 0.012(-0.092,0.116) | 0.069(-0.021,0.159) |
| MCNP | -0.115(-0.215,-0.015) | -0.120(-0.257,0.017) |
| MCOP | 0.144(0.030,0.259) | 0.139(0.007,0.270) |
| MnBP | -0.035(-0.207,0.138) | -0.210(-0.410,-0.009) |
| MCPP | -0.149(-0.268,-0.030) | 0.034(-0.103,0.171) |
| MEP | -0.027(-0.108,0.053) | 0.039(-0.071,0.150) |
| MiBP | 0.033(-0.099,0.165) | 0.069(-0.115,0.252) |
| MBzP | 0.068(-0.027,0.163) | -0.060(-0.214,0.094) |

Values in bold are statistically significant *(P* _FDR_ < 0.05*)*

Note: The total concentration of di (2-ethylhexyl) phthalate (∑DEHP) was estimated using the sum of the molar concentrations of MECPP, MEHP, MEHHP, and MEOHP and multiplied by the molar weight of MEHP (MW=278). *P* _FDR_ is the *P* value adjusted by the method of Benjamini-Hochberg false discovery rate (FDR) correction to adjust for multiple testing. All models were adjusted for age, gender, race, education, BMI, energy, protein intake, and urinary iodine.
